# Alzheimer’s Disease and Toxins Produced by Marine Dinoflagellates: An Issue to Explore

**DOI:** 10.3390/md20040253

**Published:** 2022-04-02

**Authors:** Maria João Botelho, Jelena Milinovic, Narcisa M. Bandarra, Carlos Vale

**Affiliations:** 1 IPMA, Portuguese Institute for the Sea and Atmosphere, Av. Alfredo Magalhães Ramalho 6, 1495-165 Algés, Portugal; mjbotelho@ipma.pt; 2 CIIMAR, Interdisciplinary Centre of Marine and Environmental Research, University of Porto, Terminal de Cruzeiros do Porto, Av. General Norton de Matos s/n, 4450-208 Matosinhos, Portugal; carlos.vale@ciimar.up.pt; 3 CIQ-UP, Chemistry and Biochemistry Department, Faculty of Sciences of University of Porto, Rua do Campo Alegre s/n, 4169-007 Porto, Portugal; j.milinovic@fct.unl.pt

**Keywords:** Alzheimer’s disease, marine dinoflagellates, phycotoxins, β-amyloid plaques, hyperphosphorylated tau protein

## Abstract

This paper examined the toxins naturally produced by marine dinoflagellates and their effects on increases in β-amyloid plaques along with tau protein hyperphosphorylation, both major drivers of Alzheimer’s disease (AD). This approach is in line with the demand for certain natural compounds, namely those produced by marine invertebrates that have the potential to be used in the treatment of AD. Current advances in AD treatment are discussed as well as the main factors that potentially affect the puzzling global AD pattern. This study focused on yessotoxins (YTXs), gymnodimine (GYM), spirolides (SPXs), and gambierol, all toxins that have been shown to reduce β-amyloid plaques and tau hyperphosphorylation, thus preventing the neuronal or synaptic dysfunction that ultimately causes the cell death associated with AD (or other neurodegenerative diseases). Another group of toxins described, okadaic acid (OA) and its derivatives, inhibit protein phosphatase activity, which facilitates the presence of phosphorylated tau proteins. A few studies have used OA to trigger AD in zebrafish, providing an opportunity to test in vivo the effectiveness of new drugs in treating or attenuating AD. Constraints on the production of marine toxins for use in these tests have been considered. Different lines of research are anticipated regarding the action of the two groups of toxins.

## 1. Introduction

Alzheimer’s disease (AD) is the most common irreversible neurodegenerative disease and is manifested by a progressive lack of memory and the presence of cognitive disorders. AD has become a big concern for society since more than 50 million cases have been registered worldwide, and the prevalence is forecast to increase (up to 132 million by 2050) with increased life stress [1]. AD affects mostly the elderly (over 65 years old) population but can also develop with early-onset (up to 10%) as familial AD (fAD). The development of fAD is linked to different genetic mutations and can have fatal outcomes [1,2,3,4]. The population with early symptoms of AD develops dementia as a function of time while waiting for adequate treatment [5]. As the recent pandemic globally expanded, it was found that patients infected by SARS-CoV-2 had a fivefold increase in fatal outcomes due to AD [6,7].

AD occurs via a complex pathophysiological mechanism and results in neuronal death, the loss of synapses and cholinergic neurons, brain damage, and ultimately, cell death [8,9]. This disease progresses with an increase in β-amyloid plaques, along with the hyperphosphorylation of tau proteins that gives rise to neurofibrillary tangles that cause the progressive degeneration of neurons [10]. Despite the levels of proteins in cell membranes and acetylcholine (ACh) in neurons [11], the key reason for AD development is not well understood. A challenging task is therefore to discover an adequate therapy for AD. Up to now, there are no available drugs capable of halting the disease’s progression, but alternatively there are those that are capable of mitigating the symptoms [12]. Medications for AD aim to improve cognition and relieve behavioral symptoms, although many approved drugs provide only modest benefits for the patient [13,14]. Presently, only several drugs have been approved for AD treatment, five in the US and four in the EU. Memantine (an antagonist of the N-methyl-D-aspartate receptor) and rivastigmine, galantamine, and donepezil (inhibitors of acetylcholinesterase, AChE) were approved by the US Food and Drug Administration (FDA) from 1996 to 2006 and are in use in both regions [15]. Other therapeutic options containing a fixed-dose combination of memantine and donepezil received approval in 2014 by the FDA [4]. In June 2021, aducanumab (Aduhelm™) was approved by the FDA as a novel drug for AD treatment despite polemics uttered against it by the scientific advisory committee [16]. This treatment was presented as the first therapy that targets the fundamental pathophysiology of the disease since it is based on the surrogate endpoint of the reduction in amyloid-beta plaque in the brain [16,17]. In 2019, a drug in China, sodium oligofructose (GV-971), was approved for the treatment of AD in patients with mild to moderate cognitive dysfunction [18].

The current pipeline of drugs in clinical trials for the treatment of AD is summarized in Cummings et al. [19]. They pointed to 126 agents in assessing new therapies for AD, and the large majority of drugs in clinical trials target the underlying biology of AD with the intent of disease modification. To reduce the side effects of AD, a new wave of treatments is based on natural products with neuroprotective properties and that have fewer side effects compared with synthetic drugs as they are extracted from flora and fauna [4,20,21,22]. Additionally, musicotherapy is becoming popular because behavioral studies have shown that music can improve some cognitive functions in AD patients [23]. El Haj et al. [24] suggest that music can be used as a cue to evoke involuntary autobiographical memories and emotional responses. Studies have provided evidence that neurodegenerative trajectories may be modifiable through late-life physical exercise [25], which can be considered as an alternative AD therapy.

Several studies have pointed to marine species such as bacteria, algae, sponges and invertebrates as potential sources of products that contest or slow down AD [4,26,27,28,29]. These species can synthesize several classes of metabolites to immobilize and capture prey and defend against predators [4,30]. Algae are among the most promising of these organisms [9,31,32,33,34]. A few microalgal species and bacteria naturally produce chemical compounds designated as marine toxins due to their toxic actions in some organisms [35,36,37]. In particular, through accumulation in the food chain, these toxins may be transferred to humans. Following the consumption of marine products with high concentrations of toxins, consumers may develop diarrhea, amnesia, paresthesia, or other neurological symptoms [38]. The risk of toxicity is higher in shellfish due to the high filtration rates of these organisms [39]. In parallel with the toxicity to shellfish consumers, selected toxins have been identified as promising pharmacological effectors of neurodegenerative diseases associated with memory impairment. For example, okadaic acid (OA) may cause the inhibition of protein phosphatases 1 and 2A (PP2A), which result in the hyperphosphorylation of tau protein [40,41]. Yessotoxin (YTX), gymnodimine (GYM), 13-desmethyl spirolide-C (SPX 1), and gambierol are associated with the attenuation of tau protein hyperphosphorylation and β-amyloid plaques [42,43,44,45]. These observations encourage consideration of the hypothesis that marine toxins may contribute to therapies for AD [46,47].

The present paper reviews the available literature relating to AD and toxins produced by marine dinoflagellates and examines the different impacts on the mechanisms triggering the attenuation of β-amyloid plaques along with the hyperphosphorylation of tau proteins or those that induce changes resulting in the development of similar conditions to those in AD pathology.

## 2. Alzheimer’s Disease Worldwide and a Discussion of Major Influencing Factors

Based on worldwide statistical data [48], a rank of countries with fatal outcomes due to AD in developed and developing countries can be observed (Table 1). For example, a few countries in Europe (Finland, the United Kingdom, and the Netherlands), most of the countries in North and West Africa (e.g., the Maghreb region, Angola, and Namibia) and in Asia (e.g., the Arabian Peninsula, China, and Indonesia) have a very high risk of fatal outcomes, with fatality rates between 33 (Bahrain) and 58 (Turkey) per 100,000 inhabitants. High fatality rates are present in the United States and both Central and Southern Africa. These intervals contrast with the lower mortality rates registered in most countries in Europe and South America, which have medium and low fatality rates (between 18 and 25, and lower than 17, respectively, per 100,000 inhabitants,).

### Possible Factors Influencing the Geographic Variability of Alzheimer’s Disease

The high variability of fatal outcomes among neighboring countries (e.g., the Scandinavian countries and Finland) indicates the co-existence of several factors influencing the risk of developing AD. It is generally accepted that one of the most important factors in AD is the total size of the elderly population per country. To assess the relevance of this factor, the sizes of the elderly populations in the affected regions were compared. According to the database of the Population Reference Bureau (PRB) [49], China, India, the United States, Japan, and Russia are the top five countries on the list with the largest elderly populations (21.4–166.4 million). Of these five countries, only China shows a very high rate of fatal AD outcomes, while the other four countries generally have low rates. In Europe, Germany, Italy, and France have the highest populations of elderly people (13.2–17.8 million) and have low fatal AD outcomes. Two countries in Europe with very high fatality rates, the United Kingdom and the Netherlands, have elderly populations of 12.2 and 3.3 million, respectively [49]. The case of Finland is particularly interesting, as this country has the highest rate of fatal outcomes due to AD in Europe but is not listed in the top 50 countries in terms of elderly population size. This simple example shows the lack of a positive linear trend for AD fatal outcomes according to elderly population size.

Most likely, other than socio-demographic factors and genetics, some other factors can influence this distribution. Among them, ecological and climate conditions can be of particular importance because they can result in housing structures harboring molds and the development of several species of fungi (e.g., *Aspergillus*, *Penicillium*, and/or *Stachybotrys* spp.) capable of producing neurotoxic mycotoxins [50]. Exposure to mycotoxins may also occur through food consumption (e.g., plant-food supplements) that may contain ochratoxin, produced by some *Aspergillus* species [51]. In line with the specificity of certain regions that may contribute to the geographical variability of AD are the cyanobacteria found in the mollusks and fish in the Baltic Sea and blooms in the gulfs and lakes of Finland, Canada, and the United States; such bacteria produce the neurotoxin beta-N-methylamino-L-alanine (BMAA), known to cause dementia and related disorders [52,53].

Environmental contamination by elements such as Cd, Hg, and As, which can contribute to neurotoxicity, may be another risk factor in AD development [50]. Moreover, although the relation between Al and AD has long been studied, there is no consensus on the role of the neurotoxicity caused by this element in the development of AD [54,55,56]. Recent epidemiological studies conducted in Canada found a positive linear trend between Al concentrations in drinking water and the risk of AD in the cohort but no overall association [57]. Epidemiological evidence on exposure to these elements and the risk of AD remains conflicting and a possible or plausible link is still a controversial issue. 

## 3. Applications of Marine Natural Products in Alzheimer’s Disease 

Natural products (NPs) extracted from marine flora and fauna offer a huge pipeline of molecules, including potential drugs and nutraceuticals with promising applications as neuroprotectors e.g., [4,30]. Several recent papers described NPs and their isolated compounds, such as alkaloids, lignins, polyphenols, and polyunsaturated fatty acids (PUFA), as having potential functions in the treatment of AD e.g., [20,21,22,58]. More than 10,000 products of biotechnological interest have been isolated from the organisms in marine trophic chains [59]. The mechanisms of action that have been identified in NP applications in AD prevention and treatment are: (i) AChE inhibitors; (ii) antioxidant properties; and (iii) anti-amyloidogenic agents [60]. 

For example, Table 2 lists some of those compounds, their chemical structures, and their major mechanisms of action in AD treatment. Anabaseine (3,4,5,6-tetrahydro-2,3′-bipyridine) is an alkaloid produced by nemertines, a phylum of carnivorous marine worms. Anabaseine was found to stimulate a wide variety of animal nicotinic acetylcholine receptors (nAChR). Cholinergic synaptic dysfunction indeed contributes to cognitive impairment in AD, in particular as a result of increased concentrations of Aβ peptides and their interactions with nAChR [4]. Betaine can be isolated from different seaweeds, seafood, or spinach and has an important role as an antioxidant which protects sulfur-amino-acid metabolism against oxidative damage [61]. Bryostatin-1 is found in the marine invertebrate bryozoan *Bugula neritina*. Bryostatins are hydrophilic structures that activate protein kinase enzymes (PKC), which control the function of other proteins and induce self-phosphorylation. These compounds are pharmacologically promising with high biological activity and have an important role in preventing the loss of and increasing the maturation of synaptic membranes [59,62]. Docosahexaenoic acid (DHA, 22:6n-3) is abundant in marine foods as the main component of neuronal membranes. DHA derivatives, termed docosanoids, are deemed as potential mediators of the biochemical processes in neuronal tissues that prevent or delay the inflammatory process related to AD [22]. The research into the role of neuroprotectin D1 indicated evidence of a neuroprotective effect [63]. Sterols are triterpenoids with a cyclic structure that are biosynthesized by seaweeds via the mevalonic acid pathway. Recent work pointed out that fucosterol (isolated from brown algae species *Sargassum horridum*) attenuates Aβ_1-42_-induced neurotoxicity, preventing Aβ_1–42_ oligomerization [64]. Another marine natural product is caulerpenyne, a sesquiterpene isolated from algae (*Caulerpa* spp). Caulerpa promotes successful lipoxygenase (LOX) inhibition, linked to the AD protection mechanism [65]. Zonarol is also a sesquiterpene (C15) isolated from brown algae (e.g., *Dictyopteris* spp.) that protects neuronal cells from oxidative stress damage [65], which is one of the main mechanisms of the phenolic compounds [66].

Other types of compounds with an important role in AD treatment are oligosaccharides, such as the unsaturated mannuronate oligosaccharide (MOS) extracted from some brown seaweeds (e.g., *Ecklonia* spp.), which inhibits Aβ aggregation and reduces Aβ levels via inhibiting the production BACE1. These data shed light on a novel application prospect for MOS as a promising functional food or a natural medication for the treatment of or assistance in the treatment of AD [67]. 

Although the compounds isolated from marine NPs have great therapeutic potential, they require more studies of their pharmacokinetic profiles. It should be noted that those compounds exhibited a wide range of molecular weights, of different configurations and symmetries varying from simple to polycyclic and polymeric structures, and of various functional groups, such as hydroxyl, methyl, and carbonyl groups (Table 2). 

## 4. Potentialities of Toxins Produced by Marine Dinoflagellates 

Similar to NPs extracted from flora and fauna, there are bioactive compounds in phytoplankton species such as dinoflagellates, with different chemical structures and functions as a result of their wide range of functional groups and toxicological and biological features. Macrolides, cyclic polyethers, cyclic imines, spirolides, and purine alkaloids are examples of those categories [68]. Due to their dissimilar functional structures, toxins produced by marine dinoflagellates may strongly affect a variety of biological receptors and metabolic processes [69], thereby becoming relevant tools in human medicine. Examples of potential pharmacological activities are analgesic, antitumor, anticholesterolemic, anti-inflammatory, cytotoxic, anti-infective, immunosuppressive, and as therapeutics in neurological disease [70].

### 4.1. Yessotoxins

Yessotoxins (YTXs) are marine sulfated polyethers produced as secondary metabolites by the dinoflagellates *Protoceratium reticulatum* [71], *Lingulodinium polyedrum* [72,73], and *Gonyaulax spinifera* [74]. YTXs are composed of a distinctive ladder shape formed by several ether rings of different sizes and a terminal acyclic, unsaturated side chain consisting of nine carbons and two sulfate ethers (Table 3). 

More than 90 YTX analogs are known, but only a few dozen have been fully identified. This group causes death in mice after intraperitoneal injection, although no human toxicity caused by the consumption of YTX-contaminated shellfish has yet been reported [74]. Moreover, only ultrastructural cardiac damage without other alterations was reported after oral, intraperitoneal, or intravenous administration to rats or mice [75,76]. 

Although the mechanism of action of YTXs has not been well characterized, the involvement of adenosine 3′,5′-cyclic monophosphate (cAMP), calcium, phosphodiesterases (PDEs), protein kinase (PKC), A-kinase anchor protein 149 (AKAP-149), and the mitochondria has been pointed to. The role of each one and the final effect appear to depend on the cellular model studied [77]. Several subtypes of PKC, a protein involved in multiple biological events, were reported to affect some metabolic pathways activated by YTX in the primary cortical neurons of mice and the mouse T-lymphocyte cell line EL-4 [45,78]. Alonso et al. [45] studied the in vitro effect of YTX against AD hallmarks and observed that pretreatment of cortical 3xTg-AD neurons with a low nanomolar concentration of YTX showed a decreased expression of hyperphosphorylated tau isoforms and intracellular accumulation of amyloid-beta. The mechanism related to the decrease was the activation and translocation to the plasma membrane of cytosolic PKC. In 2012 the patent “Use of YTX and analogues and derivatives for treating and/or preventing neurodegenerative diseases linked to tau and β-amyloid” was filed by Botana et al. [10], which is very promising in terms of the applicability of YTX in AD pathology. 

### 4.2. Gymnodimine

Gymnodimine (GYM) is a cyclic imine toxin belonging to a family of lipophilic, macrocyclic compounds with imine (carbon–nitrogen double bond) and spiro-linked ether moieties, with shared structural features such as spirolides (SPX), pinnatoxins, pteriatoxins, prorocentrolides, and portimine [79,80,81]. GYM (including Gymnodimine-A and its two analogs GYM-B and -C) is produced by the dinoflagellate *Karenia selliformis* (formerly named *Gymnodinium selliforme*) [82]. GYM-12 was isolated from *Alexandrium ostenfeldii* [83] and GYM-D was more recently found as a new analog [84]. GYM molecules typically present a six-membered cyclic imine, with no methyl substituents in the spiroimine ring system, and with typical fragments such as a tetrahydrofuran ring and unsaturated lactones [85] (Table 3). Studies have demonstrated that these compounds can target neuronal and muscular nicotinic ACh receptors with high affinity [86]. However, evidence for neurotoxicity in humans following consumption of contaminated seafood is currently not proven. Previous toxicological studies reported high acute toxicity in mice following intraperitoneal injection [86,87]. 

Alonso et al. [43] evaluated the effect of the long-term exposure of cortical neurons to GYM in the progress of AD pathology in vitro. Treatment of cortical neurons with 50 nM GYM decreased the intracellular β-amyloid accumulation and the levels of the hyperphosphorylated isoforms of tau protein were recognized by AT8 and AT100 antibodies. These results were suggested to be mediated by the increase in the inactive isoform of glycogen synthase kinase-3 (phospho-GSK-3 (Ser 9)), the decrease in the levels of the active isoform of the ERK1/2 kinase, and the increase in ACh synthesis elicited by the long-term exposure of cortical neurons to the toxin. Moreover, GYM decreased glutamate-induced neurotoxicity in vitro, which indicates that these compounds, by reducing the activity of excitatory neurotransmitters, constitute a valuable tool for the development of drugs to treat neurodegenerative diseases such as AD. Similar to YTX, in 2012 the patent “Use of gymnodimine, analogs, and derivatives for the treatment and/or prevention of neurodegenerative diseases associated with tau and β-amyloid” was filed by Botana et al. [88].

### 4.3. Spirolides

Also, the toxin 13-desmethyl spirolide-C (SPX 1) (Table 3), a cyclic imine such as GYM, was examined as a potential tool for the treatment of AD [42]. Spirolides (SPXs) are the largest group of the cyclic imines, and their production was confirmed in dinoflagellate species of the genus *Alexandrium* (*A. ostenfelii*/*A. peruvianum*) [89]. The toxicity of this group of compounds was first detected in routine toxin monitoring of bivalve mollusks when an unusually rapid mouse death after the intraperitoneal injection of lipophilic extracts of scallops and mussels revealed a highly potent toxic response in mice [90]. Presently 14 SPX analogs are known, of which 13-desmethyl SPX C is the most commonly found in shellfish. Although SPXs were detected in many countries around the world, they were not linked to human toxicity [86]. 

The mechanism of action of SPXs has not been fully elucidated yet, but cholinergic (muscarinic and nicotinic) receptors have been proposed as the main targets of these toxins [91]. Several studies evidenced that modulation of the cholinergic system influences the progression of AD [92]. Alonso et al. [42] took advantage of the described action of SPXs on cholinergic receptors to evaluate their potential use in AD treatment through the effect of SPX 1 (at non-toxic concentrations) on β-amyloid accumulation and tau hyperphosphorylation in a neuronal model from triple transgenic mice (3xTg). The obtained results showed that long-term exposure of 3xTg cortical neurons to the toxin significantly reduced the levels of the hyperphosphorylated isoforms of tau recognized by AT8 and AT100 antibodies as well as reduced the intracellular levels of β-amyloid. These decreases are in agreement with the modification of two of the main kinases involved in AD, t GSK-3 and the ERK1/2 kinases, also produced by 13-desmethyl SPX-C. In addition, the toxin showed a neuroprotective effect over the glutamate-induced toxicity joined to an increase in intracellular ACh levels, showing no effect on the expression levels of the cholinergic receptors studied [42].

### 4.4. Gambierol 

Gambierol is a marine polycyclic ether toxin (Table 3) that was first isolated with other bioactive compounds, such as maitotoxins and ciguatoxins, from cultured strains of the dinoflagellate *Gambierdiscus toxicus* [93,94]. Ciguatoxins are responsible for ciguatera seafood poisoning (CSP), the most prevalent phycotoxin-related seafood poisoning in humans across the globe, affecting between 10,000 and 50,000 people annually through the consumption of contaminated tropical and subtropical marine seafood [95]. Therefore, it has been speculated that gambierol may contribute to the symptoms observed in CSP outbreaks [96] since gambierol exhibits potent toxicity when administered intraperitoneally to mice, and the symptoms resemble those produced by ciguatoxins [97,98]. Gambierol and its tetracyclic and heptacyclic analogs exert a powerful modulatory action upon voltage-gated K^+^ channels (Kv). They act as an intermembrane anchor by binding specifically to Kv3.1 channels that, in turn, block Kv channels. As a consequence, the channels remain closed, thus lowering K^+^ ion currents [99,100]. In the patent “Use of gambierol for treating and/or preventing neurodegenerative diseases related to tau and beta-amyloid”, filed in 2012 by Botana et al. [89], it is mentioned that in AD, the potassium channels, specifically the kV3.1 channels, are diminished, and in the examples provided in the patent, surprisingly, gambierol produces the blockage thereof despite being useful for the reduction of both the overexpression of β-amyloid and the hyperphosphorylation of tau proteins. In addition, in a study by Alonso et al. [47], the effect of the tetracyclic analog of gambierol was tested in vivo in 3xTg-AD mice (10 months old) after 1 month of weekly treatment with 50 μg kg^−1^. The toxin used elicited a decrease in amyloid β_1–42_ levels and a dose-dependent inhibition of β-secretase enzyme-1 activity. This compound also reduced the phosphorylation of tau with an increase in the inactive isoform of the glycogen synthase kinase-3β [41]. These authors claimed that the combined effect on amyloid β_1–42_ and tau phosphorylation represented a multitarget therapeutic approach for AD, which might be more effective for this multifactorial and complex neurodegenerative disease than the current treatments.

### 4.5. Okadaic Acid and Its Derivatives

Okadaic acid (OA) and its derivatives, including dinophysistoxins (DTXs) DTX1, DTX2, and DTX3, are polyether toxins (Table 3) produced by several marine dinoflagellates of the species *Dinophysis* as well as selected species of the benthic *Prorocentrum* [101,102,103]. The syndrome diarrhetic shellfish poisoning, caused by these compounds, is a gastrointestinal illness with nausea, vomiting, diarrhea, abdominal pain, headache, and fever, where all symptoms generally pass within a few days [104]. 

Studies in vitro showed that OA and DTXs are potent inhibitors of the serine/threonine protein phosphatases 1 and 2A, and their adverse effects are considered to be mediated by this activity [105]. These enzymes have been implicated in a wide spectrum of reaction cascades [106]. Blocking protein phosphatase activity results in hyperphosphorylation of many cell proteins, leading to the use of OA and its analogs as useful tools for research on cellular regulation processes, namely to study AD [107]. In the AD brain, the activity of phosphatase 2A appears to be reduced [108,109], and the downregulation of this enzyme promotes the process of tau hyperphosphorylation [110]. OA, when injected into the right lateral dorsal hippocampus area of the adult rat brain, depicts an in vivo model of AD tauopathy [40,41]. Memory impairment induced by intra-hippocampal injection of OA has been reported, accompanied by remarkable neuropathological changes, including hippocampal neurodegeneration, a paired helical filament-like phosphorylation of tau protein, and the formation of β-amyloid containing plaque-like structures [111,112]. OA also influences oxidative stress, mitochondrial dysfunction, cholinergic dysfunction, apoptotic cell death, the activation of inflammatory cascades, and neurotoxicity [46]. All of these altered functions induced by OA lead to the development of a condition similar to AD pathology. Recently, an AD model in zebrafish was established by using OA to elucidate the neuroprotective effect of lanthionine ketimine-5-ethyl ester (LKE) and the effects of GSK3β inhibition by 4-benzyl-2-methyl-1, 2, 4-thiadiazolidine-3, 5-dione (TDZD-8) in the context of an AD-like condition [113,114]. LKE and TDZD-8 treatments given simultaneously with OA were able to protect zebrafish against OA-induced AD pathology. In particular, in vivo studies showed that the administration of TDZD-8 restored cognitive functions, reduced mortality, increased the expression and activity of PP2A, decreased the activity of GSK3β, and reduced the expression of tau proteins.

## 5. Challenges to Alzheimer Disease Therapies from Toxins Produced by Marine Dinoflagellates 

Toxins such as YTXs, GYM, SPX1, Gambierol, and OA and its derivatives are produced in the marine environment by a limited number of dinoflagellates. Various species of *Dinophysis* and *Prorocentrum* are producers of OA. The blooming of the toxin producers in seawater is triggered by a complex combination of favorable oceanographic conditions and the succession of phytoplankton species that are still poorly understood [115,116]. The potential use of these compounds as tools to search for new therapeutic approaches, or even as drugs to treat neurodegenerative diseases such as AD, is compromised by the insufficient quantities of dinoflagellate-generated material produced during blooms. Assuming that 150 g of a pure bioactive compound is necessary for typical preclinical studies and clinical trials [117], harvesting toxins from natural blooms thus seems impractical. Moreover, only scarce quantities of the toxins produced by dinoflagellates are commercially available and prices can range from 1000 up to 500,000 EUR per mg^−1^ (dry weight), depending on the source and purity [101]. 

The most plausible approaches for sustainable marine-toxin production for neurodegenerative and AD therapy seem to be (i) chemical synthesis and (ii) large-scale cultivation of producer organisms [117].

### 5.1. Chemical Synthesis of Marine Toxins

Despite the structural complexity of most toxins (Table 3), chemical synthesis of some toxins has been attempted [118,119,120,121,122,123]. Until now, most of the approaches were considered unsuitable as the biosynthetic pathways are exceedingly complex, with lengthy processes and low yields making them economically unfeasible [124,125]. For example, the synthesis of palytoxins, another group of marine toxins, involved the assembly of 7 building blocks in 39 steps, requiring a process totaling more than 140 steps [126]. In addition, more efficient synthetic routes were proposed for some compounds, and toxin-characteristic fragments have been produced to overcome these constraints [127]. Thus, the investigation of the synthesis of OA was pursued for its derivatives due to their potential broad range of toxicological and biological activities [118,121,122]. Recently, OA appeared as a commercial product produced by chemical synthesis and available worldwide [101,117].

### 5.2. Large-Scale Cultivation of Producer Organisms 

Marine dinoflagellates are known to have complex circadian rhythms controlling their cell cycles and behaviour in vivo as they establish a vertical migration pattern according to daylight and nutrient levels in nature [127]. Cell division of dinoflagellates occurs during the dark period, while both the cell growth and biosynthesis of many toxins occur during the light phase that corresponds to G1 in the cell cycle [128]. Toxin production is considered to be dependent on growth rate rather than due to environmental stress [129]. Therefore, a compromise must be reached between high toxin production and maximum growth rate. For this purpose, these marine organisms need to use optimum nutritional requirements. 

To obtain sufficient quantities of the metabolites of interest as toxins, researchers have been investing in the development of bioreactor-controlled dinoflagellate cultures and making efforts to understand the mechanisms responsible for low biomass and low toxin productivity [130,131,132]. In comparison with eukaryotic cells, dinoflagellate cells have singular metabolic requirements that can hardly be provided for with conventional photobioreactor designs. In addition to nutritional requirements, parameters such as illumination, patterns of agitation, optimal temperature, pH, oxygen tolerance, and ionic strength are essential to optimizing toxin productivity [101].

It is well known that massive production of toxins from dinoflagellate cultures is extremely difficult since dinoflagellates grow slower than other protists such as diatoms, and they are quite shear-sensitive and relatively inefficient at nutrient uptake [133]. In general, two-step culture strategies are used, with an initial phase based on biomass accumulation under nutrient replete conditions, which is followed by toxin production under deprivation conditions [69]. 

Wang et al. [132] developed an effective method for large-volume cultivation of *Prorocentrum lima* using a vertical flat photobioreactor. The maximum *P. lima* cell concentrations and OA and DTX1 contents were reached after 35 days of cultivation. Pan et al. [128] also used carboy reactors (batch operation) to study the growth cycle and the cell cycle of the dinoflagellate *Prorocentrum lima*, obtaining the following toxins: OA, OA C8-diol-ester (OA-D8), DTX-1, and DTX-4. YTXs from a large-scale culture (226 L; carboy reactors in batch operation) of a *Protoceratium reticulatum* strain were produced showing a convenient isolation method that yielded large quantities (193 mg) of high-purity yessotoxin 1 with high recovery (77%). In addition, the toxin obtained was suitable for toxicological and immunochemical studies as well as the preparation of derivatives for structure-activity studies [133]. 

Genetic manipulation methodologies using the overexpression of specific microalgal genes involved in the toxin synthesis pathways could be an attractive approach to increase toxin production. However, current knowledge about the biochemical processes occurring within the cells is still limited, probably due to the long and complex genome sequences of microalgae [134,135].

## 6. Summary and Future Works

**Figure 1:**
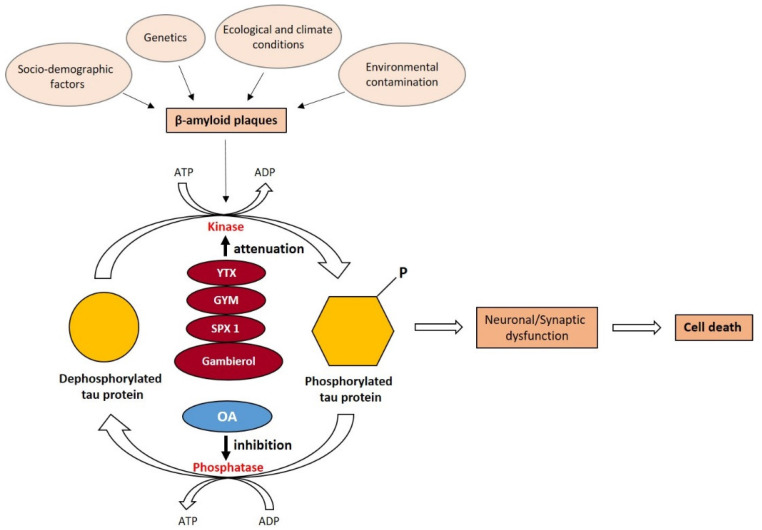
Schematic representation of molecular mechanism of AD development, including the accumulation of β-amyloid plaques and hyperphosphorylation of tau proteins and the possible impact of selected marine toxins through this mechanism.

The present study evidences the role of various toxins produced by marine dinoflagellates, such as yessotoxin, gymnodimine, 13-desmethyl spirolide-C, and gambierol, in AD pathology. The mechanisms of action of these toxins result in the decrease of β-amyloid plaques and hyperphosphorylation of tau proteins, as illustrated schematically in Figure 1. The ability of selected marine toxins to attenuate kinase observed in in vivo models encouraged researchers to consider the hypothesis that these toxins could be used in the therapy of AD or other neurodegenerative diseases. To validate this hypothesis, it is crucial to obtain sufficient quantities of toxins, either by chemical synthesis or by the cultivation of the producer dinoflagellate at an adequate scale. Then, clinical trials and detailed research are necessary with the select individuals suffering from various types of neurodegenerative diseases. 

Interestingly, OA, another toxin produced by the dinoflagellates *Dinophysis* spp. and *Prorocentrum* spp., exhibits a different mechanism of action compared with other toxins (Figure 1). By inhibiting the phosphatases, OA and its derivatives facilitate the hyperphosphorylation of tau proteins, which leads to neuronal and synaptic dysfunction and cell death. The few studies showing these effects have used OA as a model to trigger AD in vivo, thus providing the opportunity to test the effectiveness of new drugs in the treatment or attenuation of AD. The production of OA and its derivatives, either by chemical synthesis or cultivation of producer organisms, is at a more advanced stage than other toxins. Those toxins may be considered pioneer compounds in the study of the treatment of neurodegenerative diseases that are of great concern in the coming years.

## Figures and Tables

**Table 1:** Rank of countries with rate of fatal outcomes (per 100,000 inhabitants) due to the AD. Source: www.wolrdlifeexpectancy.com [48].

Classification	Country	Rate
**Very high**	Turkey	58
	Lebanon	56
	Libya	53
	Finland, Equatorial Guinea, Tunisia, Yemen, Jordan, Saudi Arabia, Morocco, Nigeria, Qatar, Iran	51–42
	Indonesia, Syria, Cambodia, Kiribati, Thailand, Laos, Timor-Leste, Mali, Myanmar, United Kingdom, Algeria	41–38
	Mauritania, Gabon, Malaysia, Gambia, Egypt, Arab Emirates, Maldives, Sri Lanka, China, Burkina Faso, Sierra Leone	37–35
	Afghanistan, Namibia, Sudan, Comoros, Togo, Angola, Netherlands, Bahrain	34–33
**High**	United States, Micronesia, Djibouti, DR Congo, Brunei, Oman, Senegal, Congo, Seychelles, Guinea, Cote d’Ivoire, Paraguay, Iraq	32–31
	South Africa, Ghana, Niger, Malawi, El Salvador, Iceland, Nicaragua, Zimbabwe, Belize, Rwanda, Tonga	30–29
	Botswana, Samoa, Sweden, Cameroon, Liberia, Mozambique, Vietnam, Chad, Benin, Cape Verde, Central Africa, Ireland, Uganda, Tanzania	28–27
	Georgia, Solomon Islands, North Korea, Peru, Vanuatu	26
**Medium**	Nepal, Eritrea, Switzerland, Suriname, Denmark, Ethiopia, Albania, Guinea-Bissau, Norway, Swaziland, Canada, Bosnia and Herzegovina, Montenegro	25–24
	Burundi, Australia, Zambia, South Sudan, Honduras, Kazakhstan, Slovakia, Somalia, Belgium, Tajikistan, Lesotho, Spain	23–22
	Armenia, Turkmenistan, Pakistan, Sao Tome, New Zealand, Cuba, Kenya, Haiti, New Guinea, Mongolia, Bolivia, Azerbaijan, France, Dominican Republic, Belarus, Madagascar, Ukraine, Jamaica	20–18
**Low**	Barbados, Bhutan, Russia, India, Luxembourg, Bahamas, Uruguay, Portugal, Germany, Cyprus, Hungary, Israel, South Korea, Italy, Malta, Bangladesh	17–14
	Chile, Czech Republic, Brazil, Serbia, Costa Rica, Austria, Croatia, Trinidad/Tobago	12–8
	Panama, Greece, Japan, Latvia, Antigua and Barbuda, Argentina, Grenada, Lithuania, Estonia, Romania, Ecuador, Saint Vincent, Guatemala, Guyana, Colombia, Moldova	6–4
	Mexico, Poland, Slovenia, Venezuela, Mauritius, Saint Lucia, Uzbekistan, Philippines, North Macedonia, Kyrgyzstan, Bulgaria	3–1
	Kuwait, Fiji, Singapore	<1

**Table 2:** Examples of marine natural products with potential functions in treatment of AD.

Source	Compound	Chemical Structure	Anti-Alzheimer Activity Mechanism	Reference
*Amphiporus* spp.	3-(2,4-Dimethoxybenzylidene)-anabaseine	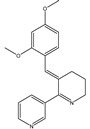	Stimulation of nicotinic acetylcholine receptors (nAChR)	[4]
Seafood Spinach Seaweeds	Betaine	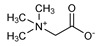	Trimethyl derivative of glycine, labile donor of methyl groups. Improve sulphur aminoacids metabolism protecting against oxidative stress	[61]
*Bugula neritina*	Bryostatin-1	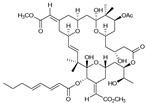	Reduces amyloid plaque. Activator of PKC revealed to prevent synaptic loss with increasing synaptic maturation	[62]
Seafood	Docosahexaenoic acid (DHA)	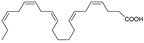	Protective against the dendritic pathology associated with expression of mutated amyloid precursor protein (APP).	[22]
*Sargassum horridum*	Fucosterol	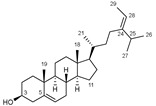	Neuroprotective agent with anti-amyloid properties	[64]
*Caulerpa racemosa*	Caulerpenyne	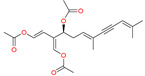	Anti-inflammatory activity	[65]
*Dictyopteris undulate*	Zonarol	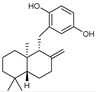	Neuroprotective activity	[65]
Brown seaweeds	Mannuronate oligosaccharide (MOS)	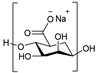	Inhibits the tau protein aggregation. Attenuates the phosphorylation of tau protein	[66]

**Table 3:** Examples of toxins naturally produced by phytoplankton species with potential functions in treatment of AD.

Source	Compound	Chemical Structure	Relation with Anti-AD Activity	Reference
*Protoceratium reticulatum*; *Lingulodinium polyedrum*; *Gonyaulax spinifera*	Yessotoxin	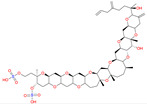	Reduces amyloid plaque and the tau hyperphosphorylation	[45]
*Karenia selliformis*	Gymnodimine	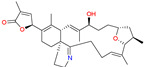	Reduces amyloid plaque and the tau hyperphosphorylation	[43]
*Alexandrium ostenfelii;* *A. peruvianum*	13-desmethyl spirolide C	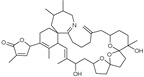	Reduces the tau hyperphosphorylation; Neuroprotective activity	[42]
*Gambierdiscus toxicus*	Gambierol	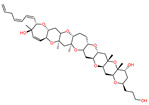	Inhibits the voltage-dependent Na and K channels; Reduces the tau hyperphosphorylation and the overexpression of B-amyloid	[47]
*Dinophysis acuta*; *D. acuminata*; *D. caudata*; *D. fortii*; *D. infundibulum*; *D. miles*; *D. norvegica*; *D. ovum*; *D. sacculus*; *Prorocentrum arenarium* *P. belizeanium*; *P. concavum* *P. lima*	Okadaic acid	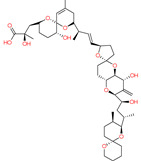	Inhibits the serine/threonine protein phosphatases 1 and 2A	[40,41]
